# The Effect of Hesperidin on Inflammatory Response and Oxidant–Antioxidant Systems in Benzo[a]pyrene-Exposed Non-Small Cell Lung Cancer (A549) Cells

**DOI:** 10.3390/molecules31142548

**Published:** 2026-07-22

**Authors:** Ahmet Büyükben

**Affiliations:** Program of Chemical Technology, Çay Vocational School, Afyon Kocatepe University, Afyonkarahisar 03200, Turkey; buyukben@aku.edu.tr

**Keywords:** A549, hesperidin, benzo(a)pyrene, oxidative stress, inflammation

## Abstract

Lung cancer represents a substantial global oncology burden, exhibiting mortality rates that surpass those of prostate, pancreatic, and breast cancers. Extensive research has established a correlation between exposure to polycyclic aromatic hydrocarbon mixtures—particularly those containing benzo(a)pyrene (BaP)—and elevated risks of pulmonary and dermal malignancies across various species, including humans. Hesperidin (HSP), a prominent bioactive flavonoid found in citrus fruits and medicinal herbs such as *Hypericum perforatum*, is recognized for its diverse pharmacological properties. The present study aimed to elucidate the antioxidant and anti-inflammatory efficacy of HSP against BaP-induced toxicity in the A549 non-small cell lung cancer (NSCLC) cell line. Following the determination of application concentrations via MTT assay, the modulatory effects of HSP on cellular proliferation, oxidative stress markers (TAS, TOS, and OSI), and key pro-inflammatory cytokines (TNF−α, IL−1β, and TGF−β) were systematically evaluated. Isolated exposure to BaP predominantly triggered a targeted upregulation of IL-1β; however, hesperidin demonstrated unexpected pro-oxidant dynamics, characterized by a substantial drop in TAS alongside a concurrent elevation in both TOS and OSI profiles, especially at maximum-dose concentrations. Notably, combining BaP with this heightened hesperidin regimen manifested the most severe oxidative distress phenotype, implying a synergistic pro-oxidant cascade between the two agents. On the contrary, minimized concentrations of hesperidin exerted explicit cytoprotective mitigation against the BaP-induced surge in IL-1β, thereby confirming a highly delicate and narrow therapeutic index for this flavonoid.

## 1. Introduction

Lung cancer remains the primary cause of oncology-related mortality globally. In 2019, approximately 24 million cancer cases and 10 million deaths were recorded worldwide, with lung cancer accounting for 15–20% of this total. Histologically, lung cancer is categorized into two broad classes: small cell lung carcinoma (SCLC) and NSCLC, the latter of which constitutes the majority of cases. NSCLC is further subdivided into adenocarcinoma, squamous cell carcinoma, and large cell carcinoma, each characterized by distinct therapeutic requirements and clinical outcomes [1,2]. The prevalence of lung cancer is notably higher in individuals with head and neck malignancies, a phenomenon described as the “field effect.” While tobacco consumption remains the primary etiological driver, factors such as genetic predisposition, malnutrition, occupational hazards, and atmospheric pollution also play significant independent or synergistic roles in shaping the epidemiology of the disease [3].

Tobacco-specific nitrosamines, such as 4-(methylnitrosamino)-1-(3-pyridyl)-1-butanone (NNK), and polycyclic aromatic hydrocarbons (PAHs), most notably BaP, are recognized as potent initiators of carcinogenesis [4]. BaP, a potent member of the PAHs, is a ubiquitous industrial and environmental pollutant. Originating primarily from the incomplete combustion of fossil fuels, industrial emissions, vehicle exhaust, and biomass burning, BaP is widely distributed across atmospheric particulate matter and environmental ecosystems [5]. Human exposure to this environmental toxicant—primarily through the inhalation of polluted air—poses a severe public health risk. At the cellular level, the toxicity of BaP is intrinsically linked to oxidative stress chemistry. Upon metabolic activation in the respiratory epithelium, BaP generates highly reactive intermediates and excessive amounts of reactive oxygen species. This pollutant-induced oxidative burst disrupts redox homeostasis, ultimately driving the inflammatory cascades and cellular damage characteristic of environmental PAH exposure [6]. BaP is classified as a human carcinogen based on robust and consistent evidence from both clinical and experimental models. Extensive research has established a direct correlation between BaP exposure—via various administration routes—and elevated risks of pulmonary and dermal malignancies [7]. Given its prevalence in cigarette smoke and its formation during the combustion or grilling of organic matter, human exposure to BaP is ubiquitous. The metabolic activation of this xenobiotic, mediated by the cytochrome P450 (CYP P450) system, generates 7β,8α-dihydroxy-9α,10α-epoxy-7,8,9,10-tetrahydrobenzo[a]pyrene (BPDE). This highly reactive metabolite facilitates the formation of DNA adducts, subsequently driving mutations and malignant transformations [5]. Beyond direct DNA damage, the resulting reactive oxygen species (ROS) contribute to epigenetic and non-genotoxic alterations, triggering the upregulation of stress-response genes and antioxidant defense mechanisms [8]. Furthermore, BaP exhibits epigenotoxic, neurotoxic, and teratogenic properties, alongside a marked pro-oxidative potential that impairs fertility in animal models [5].

Historically, natural products have served as the cornerstone of pharmacological drug discovery, providing structurally diverse and highly complex molecular scaffolds that are difficult to synthesize de novo [9]. In the field of oncology and toxicology, phytochemicals—particularly plant-derived secondary metabolites—are extensively investigated as primary sources of novel chemo-preventive and therapeutic agents due to their multi-targeted mechanisms of action and generally favorable toxicity profiles [10].

**Figure 1:**
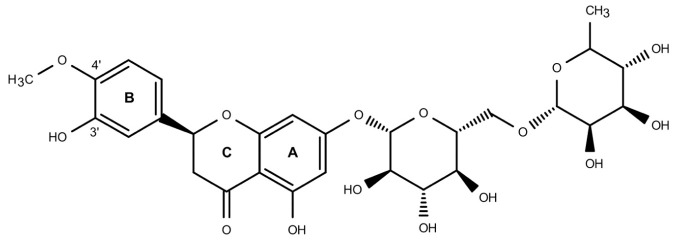
Hesperidin. The chemical structure of hesperidin was generated using FreeChemDraw “https://freechemdraw.com/ (accessed on 7 July 2026)”.

The *Citrus* genus, comprising grapefruit, lemon, orange, and tangerine, is characterized by a diverse array of edible and medicinal fruits. A primary bioactive constituent of these fruits is the flavonoid hesperidin (HSP/HSD; hesperetin 7-rutinoside), a prominent member of the polyphenol family. Hesperidin (Figure 1) is widely recognized for its multifaceted biological activities, including antioxidant, anti-inflammatory, antimicrobial, and anticarcinogenic effects [11,12,13,14]. Clinically, it has been utilized to promote the healing of venous ulcers and to reduce capillary fragility and permeability [15].

Among the vast array of bioactive phytochemicals, hesperidin emerges as a particularly compelling candidate for mitigating chemical-induced cytotoxicity. The structural selection of hesperidin over other flavonoids is highly deliberate. Structurally, it consists of an aglycone core (hesperetin) conjugated to a rutinose disaccharide. This specific glycosidic linkage significantly enhances its hydrophilicity and bioavailability compared to many other highly hydrophobic flavonoid aglycones [16]. Furthermore, the unique substitution pattern on its B-ring (a methoxy group at C-4′ and a hydroxyl group at C-3′) grants the molecule exceptional electron-donating capacity (Figure 1). This structural configuration makes hesperidin a highly potent, targeted scavenger of reactive oxygen species (ROS) and a robust modulatory agent capable of buffering the intense oxidative and inflammatory cascades typically triggered by polycyclic aromatic hydrocarbons like BaP [17,18].

The present study aimed to evaluate the therapeutic potential of hesperidin against BaP-induced toxicity in the A549 NSCLC cell line. Following the determination of application concentrations of hesperidin via MTT assay, the study systematically investigated the modulatory effects of hesperidin on cellular proliferation, oxidative stress parameters (TAS, TOS, and OSI), and key pro-inflammatory cytokines (TNF−α, IL−1β, and TGF−β).

## 2. Results and Discussion

**Figure 2:**
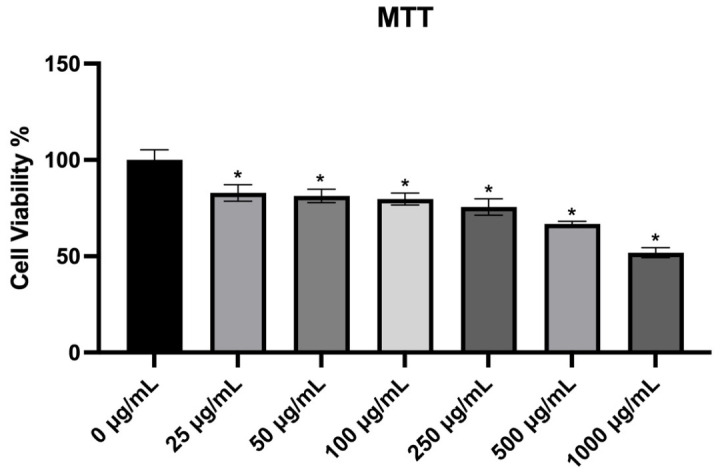
BaP-exposed A549 cell viabilities of hesperidin concentrations. *; Statistically different from the control group (0 μg/mL) (*p* ˂ 0.05). Data are expressed as mean ± SEM (*n* = 7).

MTT analysis across a range of 25 to 1000 μg/mL confirmed a concentration-dependent decline in cell viability (Figure 2). Notably, a significant reduction was observed at concentrations exceeding 25 μg/mL, aligning with the existing literature that high-dose hesperidin treatment accelerates cellular destruction [19,20,21]. MTT analysis conducted on A549 cells treated with BaP and varying concentrations of hesperidin established an IC_0_ dose of 0.128 μg/mL (0.210 μM) and an IC_50_ dose of 2367 μg/mL (3876 μM) (Table 1). The IC_50_ thresholds of hesperidin against A549 and other malignant lineages exhibit a broad variation across the literature. For instance, a study focusing on the suppression of cell proliferation and apoptosis via the downregulation of the β-catenin/c-Myc axis reported an IC_50_ value of 12.5 μM [21]. Conversely, an investigation into transforming growth factor β1-induced epithelial–mesenchymal transition via cytoplasmic Smad signaling yielded an IC_50_ of 338.9 μM after 24 h of exposure [22]. Furthermore, when exploring the antitumor efficacy of hesperidin against non-small cell lung cancer lines by targeting the miR-34a/PD-L1/NF-κB pathway in comparison with cisplatin, the IC_50_ was determined to be 814.36 μM [23]. Although the IC_50_ value observed in the present study is relatively higher than certain values documented in the literature, this discrepancy must be evaluated considering that this experimental model utilized BaP-pretreated A549 cells. BaP exposure inherently induces severe oxidative stress on the mitochondrial membrane and disrupts the respiratory chain, thereby altering the baseline metabolic rate of these cells compared to their untreated counterparts. Mechanistically, BaP ligates to the aryl hydrocarbon receptor (AhR), driving the upregulation of *CYP1A1*, which subsequently metabolizes BaP into BPDE—the ultimate mutagenic carcinogen that forms DNA adducts [24].

Oxidative stress, fundamentally characterized by a disequilibrium between the generation and scavenging of ROS and other free radicals, is inextricably linked to the pathogenesis of numerous diseases, predominantly cancer [25,26,27]. While ROS are endogenously synthesized as routine byproducts of cellular metabolism and further modulated by exogenous stimuli [28], their intracellular concentrations diverge significantly between physiological and pathological states. Specifically, basal ROS levels in healthy cells are tightly regulated at approximately 0.02 μM, whereas neoplastic cells inherently exhibit drastically elevated concentrations ranging from 100 μM to 1 mM. Consequently, quantifying oxidative stress serves as a pivotal diagnostic metric for evaluating the extent of malignancy [29,30]. Interestingly, while moderate ROS elevation is established to be pro-tumorigenic by driving proliferation cascades, supraphysiological ROS accumulation (extreme oxidative stress) paradoxically induces severe cytotoxicity [31]. To comprehensively elucidate this cellular redox dynamic, assessing the Total Antioxidant Status (TAS) and Total Oxidant Status (TOS)—which represent the cumulative pools of antioxidants and oxidants in intra- and extracellular fluids, respectively—provides significantly greater diagnostic value than quantifying individual redox molecules. Furthermore, the Oxidative Stress Index (OSI), formulated as the ratio of TOS to TAS, serves as a robust indicator of the overall oxidative burden within the biological specimen [27].

**Figure 3:**
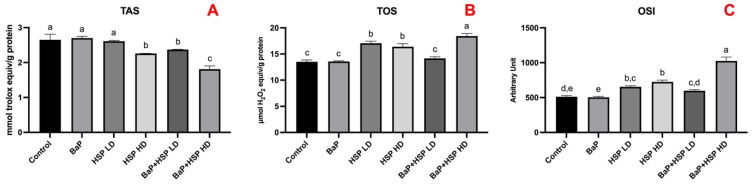
Oxidative stress parameters of the experimental groups (**A**) Total Antioxidant Capacity, (**B**) Total Oxidant Capacity and (**C**) Oxidative Stress Index. ^a.b.c.d.e^: Differences between means with different superscript letters in the same analysis are statistically significant (*p* < 0.05). There is no difference between groups with one or more of the same letters. Data are expressed as mean ± SEM (*n* = 3).

As delineated in Table 2 and Figure 3, the evaluation of TAS, TOS, and OSI parameters revealed distinct redox alterations across the experimental cohorts. Notably, no statistically significant divergence was observed between the negative control and BaP-exposed groups regarding TAS and OSI levels. In contrast, the treatment of hesperidin precipitated a marked, statistically significant reduction in TAS exclusively in HSP HD, BaP+HSP LD and BaP+HSP HD groups (*p* < 0.05), whereas HSP LD group maintained TAS levels statistically comparable to the control (*p* > 0.05). Strikingly, BaP+HSP HD group exhibited the lowest recorded TAS level (Figure 3A,C). Concurrently, while TOS levels remained relatively static between the control and BaP cohorts, a statistically significant elevation was manifested in BaP+HSP HD group, which reached the highest overall TOS magnitude. Correspondingly, significant exacerbations in TOS levels were also corroborated in HSP LD and HSP HD groups, indicating a pronounced induction of oxidative insult (Figure 3B).

The literature documents that high-dose BaP exposure in murine models exacerbates lipid peroxidation while elevating aryl hydrocarbon hydroxylase, carcinoembryonic antigen, and alanine transaminase levels, concomitantly depleting enzymatic antioxidant reserves. Hesperidin supplementation has been shown to effectively ameliorate these perturbations, substantiating its chemopreventive potential against chemically induced pulmonary carcinogenesis [32]. Parallel to this, an investigation evaluating the anticancer efficacies of thymoquinone, caffeic acid phenethyl ester, and resveratrol in BaP-exposed A549 cells reported no significant alterations in TAS and OSI parameters between the BaP and control groups [33]. Furthermore, hesperidin was demonstrated to suppress MCF-7 cell proliferation in a concentration-dependent manner, a phenomenon accompanied by a marked reduction in intracellular antioxidants. This suggests that the induction of apoptosis in these neoplastic cells is mechanistically driven by this targeted depletion of the antioxidant defense system [34].

In the present experimental paradigm, A549 cells were treated with BaP, which is a potent pulmonary procarcinogen, an already inherently malignant lineage. Similar to the study by Ulasli et al. [33], no statistically significant divergence was observed in TAS, TOS, or OSI levels between the negative control and BaP-only cohorts. This outcome is mechanistically anticipated, as the BaP challenge was introduced to a cellular microenvironment already burdened by a basal hyper-oxidative state. Aligning with Natarajan et al. [34], data of the study reveal a robust dose-dependent dynamic: escalating hesperidin (HSP) concentrations synchronize with diminished cell proliferation, depleted TAS levels, and amplified oxidative stress. Notably, the BaP + HD cohort exhibited the highest TOS and OSI magnitudes coupled with the most severe TAS depletion, indicating extreme oxidative vulnerability. While the BaP + LD cohort displayed the most stabilized oxidative parameters among the HSP-treated groups, it still harbored an elevated stress load relative to the control. Consequently, these findings postulate that HSP treatment, particularly at the HD threshold, inflicts a critical oxidative insult on A549 cells, serving as a primary driver for its anti-proliferative efficacy. A similar situation is observed at the LD threshold, although the efficacy at lower doses appears to be more limited. While hesperidin is conventionally classified as an antioxidant, it is well-documented that flavonoids frequently exhibit paradoxical pro-oxidant behavior in malignant microenvironments. Due to the high basal ROS and altered transition metal metabolism inherent to cancer cells, hesperidin drives excessive intracellular ROS generation, deliberately overwhelming the neoplastic redox balance [34]. While increased oxidative stress in cancer cells is generally a therapeutically desirable and positive outcome, agents specifically used to trigger or modulate oxidative stress, such as hesperidin, should also be investigated concurrently in healthy cell lines.

The etiology of most malignancies is intricately linked to environmental and acquired factors that sustain chronic inflammatory cascades [35]. While acute inflammation acts as the primary host defense against carcinogens [36], an impaired or dysregulated inflammatory response significantly facilitates tumor progression. In this context, the potent anti-inflammatory capacity of hesperidin is well-documented, evidenced by its ability to abrogate the secretion of inflammatory mediators in allergic asthma models [37].

Inflammation is inextricably linked to the progression and development of malignancies. Tissue homeostasis relies on a delicate equilibrium between pro- and anti-inflammatory mechanisms; however, an aberrant disruption of this balance is frequently coupled with tumorigenesis within an inflammatory milieu. While specific cytokines and chemokines are canonically secreted by immune cells, malignant cells actively synthesize these mediators to orchestrate a pro-tumorigenic inflammatory microenvironment [38].

BaP drives cancer progression by enhancing the migratory and invasive capacities of A549 lung cancer cells. Mechanistically, this is mediated through the upregulation of pro-inflammatory chemokines, such as IL-8, CCL2, and CCL3, alongside the overexpression of specific regulatory genes like *TWIST1* and *LINC00673* [39]. Chemokines constitute a vast family of low-molecular-weight proteins critical for directing leukocyte chemotaxis and maintaining immunological homeostasis. Notably, CC chemokine ligand 2 (CCL2) acts as a master regulator of the immune response. Non-immune lineages, including smooth muscle cells, endothelial cells, and fibroblasts, also secrete CCL2, particularly upon stimulation by IL-6, TNF-α, and TGF-β [40]. In chronic inflammation, TNF-α acts as a pivotal pro-inflammatory cytokine that profoundly triggers CCL2 production—specifically from macrophages and endothelial cells—thereby facilitating monocyte adhesion and extravasation into inflamed tissues. This cross-talk is critical in the pathogenesis of inflammatory conditions such as Parkinson’s disease, nephropathies, and cancer-associated dermatomyositis [41].

TGF-β is s markedly overexpressed across diverse malignancies, including lung adenocarcinoma, positioning the TGF-β signaling axis as a critical driver of tumor evolution [42]. This pathway governs pleiotropic cellular responses—spanning embryonic development to tissue homeostasis—via canonical and non-canonical cascades. Dysregulated TGF-β signaling fundamentally promotes extracellular matrix deposition, culminating in fibrosis, and actively induces epithelial–mesenchymal transition, immunosuppression, and neovascularization in advanced carcinomas, thereby accelerating disease progression. Given its pathophysiological involvement in conditions ranging from inflammatory diseases to cardiovascular disorders, targeting the TGF-β cascade has emerged as a highly promising therapeutic strategy [43].

**Figure 4:**
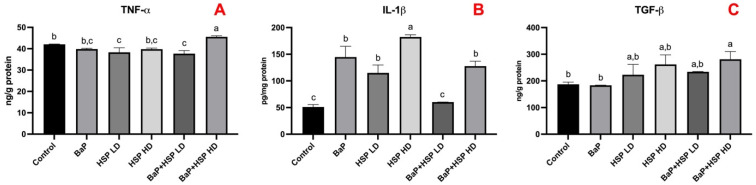
Inflammation parameters of the experimental groups (**A**) TNF-α levels (**B**) IL-1β levels (**C**) TGF-β levels. ^a.b.c^: Differences between means with different superscript letters in the same analysis are statistically significant (*p* < 0.05). There is no difference between groups with one or more of the same letters. Data are expressed as mean ± SEM (*n* = 3).

As delineated in Table 3 and Figure 4, the modulation of cytokine profiles (TNF-α, IL-1β, and TGF-β) further elucidates this dynamic. Regarding TNF-α, BaP+HSP HD group demonstrated the highest concentration, diverging significantly from all other cohorts. Conversely, HSP LD and BaP+HSP LD groups exhibited a statistically significant downregulation in TNF-α compared to the control group, whereas the BaP-only cohort showed no significant difference (Figure 4A). For IL-1β, the control group maintained significantly lower levels than all other groups except BaP+HSP LD group, while HSP HD group peaked with the highest IL-1β expression, differentiating it statistically from the rest (Figure 4B). Finally, the evaluation of TGF-β levels revealed a similar zenith in BaP+HSP HD group, presenting a significant disparity when compared to both the control and BaP groups. No statistically significant variations in TGF-β were detected among the remaining experimental cohorts relative to the baseline controls (Figure 4C).

IL-1β emerged as the most responsive inflammatory marker, showing dramatic elevations with BaP exposure and high-dose hesperidin. This finding aligns with established literature demonstrating IL-1β’s central role in BaP-induced inflammation [44]. IL-1β is a potent pro-inflammatory cytokine that activates NF-κB signaling, amplifies inflammatory cascades, and promotes cell proliferation in lung epithelial cells [45]. The ability of low-dose hesperidin to suppress BaP-induced IL-1β elevation suggests potential anti-inflammatory efficacy at appropriate doses, consistent with previous reports of hesperidin’s IL-1β-suppressing effects in lung inflammation models [46]. Interestingly, high-dose hesperidin alone induced even greater IL-1β elevation than BaP, suggesting that excessive hesperidin exposure may trigger inflammatory responses independent of BaP. This could involve activation of pattern recognition receptors, endoplasmic reticulum stress, or other cellular stress pathways [47]. The relatively stable TNF-α expressions observed across most treatment groups diverge from the existing literature, which documents pronounced BaP-induced TNF-α up-regulation in endothelial cells [24] and various other cell lineages [48]. This phenotypic discrepancy may be attributed to several distinct factors intrinsic to the alveolar epithelial model utilized herein. First, A549 adenocarcinomic cells may harbor divergent TNF-α regulatory networks compared to endothelial or specialized immune cells. Second, the kinetics of the cytokine response might follow a different timeline, with TNF-α secretion peaking at earlier or later time points than those monitored in the presented experimental design. Third, A549 cells might initiate potent negative feedback loops that strictly constrain downstream TNF-α translation under these specific exposure conditions. Interestingly, the modest yet discernible elevation of TNF-α in the BaP+HSP HD group implies that the concurrent accumulation of severe oxidative stress and pro-inflammatory stimuli can eventually override these homeostatic regulatory checkpoints. The lack of statistical significance for TGF-β despite a 50% increase from lowest to highest values reflects high inter-replicate variability. TGF-β is a pleiotropic cytokine with complex, context-dependent functions in lung pathology [49]. In cancer cells, TGF-β can promote epithelial–mesenchymal transition, fibrosis, and immune evasion [50]. The trend toward elevated TGF-β with hesperidin treatment, particularly in combination groups, warrants further investigation with larger sample sizes. These findings indicate that low-dose hesperidin treatment to BaP-challenged A549 cells does not elicit an aggressive pro-inflammatory response, but rather modestly maintains basal cytokine levels, distinctly contrasting with the inflammatory exacerbation induced by high-dose treatments.

Finally, it should not be forgotten that a limitation of the present study is the exclusive use of the malignant A549 cell line. While this approach effectively elucidates hesperidin’s modulatory capacity within an already established pro-inflammatory and hyper-oxidative tumor microenvironment under continuous BaP stress, it lacks a comparative baseline with healthy pulmonary epithelium. Future investigations incorporating non-cancerous lung cell lines are warranted to comprehensively map the differential toxicological and chemopreventive redox dynamics of hesperidin between healthy and neoplastic tissues. Also, larger sample sizes at the analyses would improve precision and enable detection of smaller effect sizes. Apart from these, while the study measured key biomarkers, it did not directly assess underlying mechanisms. Further comprehensive mechanistic studies are particularly warranted to better elucidate the observed pro-oxidant and pro-inflammatory effects of high-dose hesperidin.

## 3. Materials and Methods

### 3.1. Cell Culture and Maintenance

The human lung adenocarcinoma cell line, that is A549 (ATCC CCL-185) utilized in this study was procured from the Molecular Biology Laboratory stock of the Department of Molecular Biology and Genetics, Faculty of Science and Literature, Afyon Kocatepe University. A limitation of the present study is that routine in-house mycoplasma testing was not performed prior to the assays. But still, as a strict quality control measure, the cells were rigorously and continuously monitored under an inverted microscope throughout all passages. Their characteristic epithelial morphology, adherence capacity, and consistent proliferation rates were confirmed to be stable, and no signs of visible contamination or phenotypic drift were observed during the experiments.

### 3.2. MTT Cell Viability Assay and Experimental Design

Cell viability and cytotoxicity were evaluated using the MTT (3-(4,5-dimethylthiazol-2-yl)-2,5-diphenyltetrazolium bromide) colorimetric assay. This method relies on the active internalization of MTT by viable cells and its subsequent reduction into water-insoluble, blue-purple formazan crystals by mitochondrial succinate dehydrogenase. Because formazan generation strictly depends on active mitochondrial metabolism, the spectrophotometric absorbance is directly proportional to the number of viable cells [51].

200 µL of Dulbecco’s Modified Medium (DMEM) (Sigma-Aldrich, St. Louis, MO, USA) containing 10% FBS (Capricorn, Hessen, Germany), 1 mM sodium pyruvate (Sigma-Aldrich, St. Louis, MO, USA), and 2 mM L-glutamine (Sigma-Aldrich, St. Louis, MO, USA) was added to each well of 96-well F-based cell culture dishes for MTT analysis. Subsequently, 100 mL of A549 suspension, prepared at a concentration of 1 × 10^4^ cells/mL and containing an average of 2 × 10^3^ cells, was added to each well. The wells were incubated for 24 h to allow the cells to adhere to the substrate. After the cell cultures covered 70–80% of the well surface, the culture medium was replaced with fresh medium containing 20 µM BaP (C_20_H_12_; B1760, purity > 96%, Sigma-Aldrich, St. Louis, MO, USA) for 48 h. This specific concentration and pre-incubation timeframe were strictly selected based on previously optimized and validated in vitro models [33,52]. These foundational studies demonstrated that a 48 h exposure to 20 µM BaP effectively instigates robust oxidative stress and pro-inflammatory pathways in A549 cells without inducing immediate, widespread necrosis, thereby creating an optimal and reproducible baseline to assess the therapeutic modulation of secondary agents like hesperidin. BaP was first dissolved in dimethyl sulfoxide (DMSO) (Sigma-Aldrich, St. Louis, MO, USA) and then added to the culture medium to ensure the final concentration of DMSO was less than 0.1%. After 48 h of BaP pre-treatment, hesperidin (H5254, purity ≥ 80%, Sigma-Aldrich, St. Louis, MO, USA) was applied to each well at specific dose ranges (10 μg/mL–1000 μg/mL). The subsequent incubation period for hesperidin was intentionally restricted to 24 h. Given the initial 48 h BaP exposure, extending the hesperidin treatment to standard 48 or 72 h would increase the total continuous culture time to 96–120 h. Considering the doubling time of A549 cells, this restriction was essential to prevent overconfluence, nutrient depletion, and contact inhibition, which could trigger spontaneous cell death and severely confound the assay results. This specific timeframe allowed for the determination of the LD and HD while ensuring cell viability assessments remained strictly reflective of the compound interactions rather than culture environment limitations.

After the addition of the active ingredients, the cells were incubated at 37 °C under 5% CO_2_ partial pressure. After 24 h of incubation, MTT (3-4,5-dimethyl-thiazolyl-2,5-diphenyltetrazolium bromide) dye (Sigma-Aldrich, St. Louis, MO, USA), dissolved at a concentration of 5 mg/mL in phosphate buffer (PBS) (Sigma-Aldrich, St. Louis, MO, USA) with a pH of 7.4, was added to each well at a volume equal to 10% of the well volume (22 μL). The cells were incubated at 37 °C for 2–4 h. At the end of this period, the MTT dye stain was removed from the cells, and 200 μL of DMSO was added to each well and incubated for 10 min. Color change was determined at a wavelength of 540 nm using an ELISA plate reader (BioTek ELx800, Marshall Scientific, Hampton, NH, USA). Control cell viability, untreated with hesperidin, was considered 100%, and the viability rates of the experimental cells were expressed as percentages. For the cell viability assays, each experimental condition was tested in quintuplicate per plate, and the entire experiment was repeated independently at least three times.

As a result of MTT analyses performed on A549 cell lines with various doses of hesperidin, the IC_0_ dose was determined as 0.128 μg/mL and the IC_50_ dose as 2367 μg/mL. The results are presented in Table 1. The baseline non-toxic dose (IC_0_) that was used for low-dose (LD) group was directly observed from the empirical data. For the upper threshold, a mathematical extrapolation was performed using Probit regression analysis via SPSS software (version 25.0) based on the available dose–response curve. Since the extrapolated value (calculated as 2367 μg/mL) exceeded the maximum applied concentration of 1000 μg/mL, it was scientifically re-designated as the high-dose (HD) rather than a classical IC_50_. This HD concentration was strictly utilized in subsequent biochemical assays to deliberately induce and evaluate the extreme oxidative and inflammatory burden on BaP-stressed cells. After determining the doses, 6 experimental groups were designed for A549 cells treated with BaP.

Control: Vehicle control treated exclusively with equivalent volumes of solvent (DMSO < 0.1%).

BaP: Induced with 20 µM BaP to establish baseline toxicity.

HSP LD: Treated with 0.128 µg/mL HSP for 24 h.

HSP HD: Treated with 2367 µg/mL HSP for 24 h.

BaP+HSP LD: Pre-treated with BaP, followed by 0.128 µg/mL HSP for 24 h.

BaP+HSP HD: Pre-treated with BaP, followed by 2367 µg/mL HSP for 24 h.

### 3.3. Preparation of Cell Lysates

Cells were seeded into 5 separate 75 cm^2^ flasks for each group and placed in an incubator at 37 °C and 5% CO_2_ for growth. The cells were monitored daily, and the culture medium was changed every two days until 80% of the cells were confluent. Once sufficient confluent was reached, 20 µM BaP was applied to the BaP groups for 48 h. Afterwards, hesperidin and DMEM were applied to the relevant groups and incubated for 24 h. Following incubation, trypsinization/detrypsinization was performed to remove the cells from the flask substrate. After two washes to remove trypsin (Sigma-Aldrich, St. Louis, MO, USA), the cell pellet remaining at the bottom of the falcon tube was washed with PBS. Lysis buffer was added to the cells according to the type of analysis. Proteins insoluble in detergent were removed by centrifugation at 12,000× *g*, 4 °C for 10 min to obtain the supernatant (cell lysate) [52]. The protein levels in these cell lysates were determined using the Bradford method with bovine serum albumin as a standard [53].

### 3.4. Biochemical Analyses

To determine the effect of hesperidin on oxidative stress in A549 cells exposed to various doses of BaP, the total antioxidant capacity (TAS), total oxidant capacity (TOS), and oxidative stress index (OSI) levels were examined in the relevant cell lysates. First, total protein levels were measured using a commercial kit (Fluka 51254, Sigma-Aldrich, St. Louis, MO, USA) on an ELISA (BioTek ELx-800, Marshall Scientific, Hampton, NH, USA) device, and the Bradford method was used as the basis for this study [53]. TAS levels, as an oxidative stress parameter, were measured using commercial kits (RL0017, Rel Assay, Gaziantep, Turkey) employing spectrophotometric methods. Trolox was used as a standard in the range of 0.125–2 mmol to determine TAS levels. TOS levels, as an oxidative stress parameter, were measured using commercial kits (RL0024, Rel Assay, Gaziantep, Turkey) employing spectrophotometric methods. All experimental procedures were carried out in triplicate. Considering TAS and TOS levels, OSI levels were calculated according to the formula below, in accordance with the kit protocol [54]:

Oxidative Stress Index (OSI) = [(TOS/TAS) × 100]

### 3.5. Inflammatory Cytokine Analyses

The anti-inflammatory effect of hesperidin in A549 cells treated with BaP was tested using TNF-α, IL-1β, and TGF-β markers. Cytokine levels were determined in cell lysates using a multiplate reader (BioTek ELx-800, Marshall Scientific, Hampton, NH, USA) at 450 nm with specific commercial kits (E0082Hu, E0143Hu, E3051Hu, Bioassay Technology Laboratory, Shanghai, China). Data were expressed as mass per protein, ratioed to total protein values. All analyses were performed in triplicate.

### 3.6. Statistical Analyses

Statistical evaluations were executed using SPSS software (version 25.0). Normality was assessed, and normally distributed data were analyzed using one-way analysis of variance (ANOVA) followed by Duncan’s post hoc test. For non-parametric datasets, the Kruskal–Wallis test was employed, with the Mann–Whitney U test applied for subsequent pairwise comparisons against the control. To enhance statistical and visual clarity, all experimental data in the tables and figures are now expressed as mean ± Standard Error of the Mean (SEM). A *p*-value of *p* < 0.05 was deemed statistically significant. Statistical analyses were performed based on data obtained from at least three independent experiments, each conducted in triplicate or quintuplicate. Graphs were generated using GraphPad Prism version 11.0.2 (GraphPad Software, San Diego, CA, USA).

## 4. Conclusions

This comprehensive analysis of inflammation and oxidative stress parameters in A549 cells reveals complex, dose-dependent interactions between benzo(a)pyrene and hesperidin. While BaP alone primarily induced IL-1β elevation, hesperidin exhibited unexpected pro-oxidant effects, particularly at high doses, decreasing TAS and increasing TOS and OSI. The combination of BaP with high-dose hesperidin produced the most extreme oxidative stress phenotype, suggesting synergistic pro-oxidant interactions. Conversely, low-dose hesperidin showed protective effects against BaP-induced IL-1β elevation, indicating a narrow therapeutic window. Consequently, hesperidin supplementation in populations exposed to environmental pollutants like BaP should be approached cautiously, with careful attention to dosing and potential adverse interactions. Future research should focus on identifying optimal hesperidin doses that maximize anti-inflammatory benefits while minimizing pro-oxidant risks, and on elucidating the molecular mechanisms underlying these complex dose-dependent effects.

## Figures and Tables

**Table 1:** Hesperidin inhibitory concentrations in A549 cell lines treated with BaP.

	Hesperidin Inhibitory Concentrations for BaP-Treated A549 Cell Line, (μg/mL)	95% Confidence Interval	
		Low	High
IC_0_	0.128	0.002	0.937
IC_10_	11	2	25
IC_20_	68	30	112
IC_30_	259	162	454
IC_40_	812	461	2240
IC_50_	2367	1078	11,297

The value of IC_0_ (0.128 μg/mL) is subsequently referred to as Low-Dose (LD) group. The value IC_50_ (2367 μg/mL) was extrapolated via Probit regression analysis and is subsequently referred to as High Dose (HD) throughout the biochemical experiments to represent extreme hesperidin overload.

**Table 2:** TAS, TOS, and OSI levels of the experimental groups.

	TAS (mmol Trolox Equiv/g Protein)	TOS (μmol H_2_O_2_ Equiv/g Protein)	OSI (Arbitrary Unit)
Control	2.65 ± 0.16 ^a^	13.51 ± 0.34 ^c^	512 ± 17 ^d,e^
BaP	2.70 ± 0.05 ^a^	13.58 ± 0.11 ^c^	504 ± 14 ^e^
HSP LD	2.61 ± 0.02 ^a^	17.06 ± 0.40 ^b^	655 ± 15 ^b,c^
HSP HD	2.26 ± 0.01 ^b^	16.39 ± 0.59 ^b^	726 ± 25 ^b^
BaP+HSP LD	2.37 ± 0.01 ^b^	14.15 ± 0.32 ^c^	598 ± 15 ^c,d^
BaP+HSP HD	1.81 ± 0.09 ^c^	18.43 ± 0.48 ^a^	1026 ± 57 ^a^

Data are presented as mean ± standard error of the mean (*n* = 3). ^a.b.c.d.e^: Differences between means with different superscript letters in the same column are statistically significant (*p* < 0.05). There is no difference between groups with one or more of the same letters.

**Table 3:** TNF-α, IL-1β, TGF-β cytokine levels of the experimental groups.

	TNF-α (ng/g Protein)	IL-1β (pg/mg Protein)	TGF-β (ng/g Protein)
Control	42.08 ± 0.11 ^b^	51.05 ± 4.58 ^c^	187.15 ± 8.48 ^b^
BaP	39.86 ± 0.37 ^b,c^	144.75 ± 20.15 ^b^	183.59 ± 0.72 ^b^
HSP LD	38.30 ± 2.13 ^c^	114.92 ± 14.94 ^b^	223.17 ± 38.76 ^a,b^
HSP HD	39.83 ± 0.50 ^b,c^	182.335 ± 4.17 ^a^	261.79 ± 35.55 ^a,b^
BaP+HSP LD	37.70 ± 1.44 ^c^	60.235 ± 0.15 ^c^	233.96 ± 0.91 ^a,b^
BaP+HSP HD	45.55 ± 0.50 ^a^	127.73 ± 9.26 ^b^	281.07 ± 29.23 ^a^

Data are presented as mean ± standard error of the mean. ^a.b.c^: Differences between means with different superscript letters in the same column are statistically significant (*p* ˂ 0.05). There is no difference between groups with one or more of the same letters. (*n* = 3).

## Data Availability

Data will be made available on request.
